# Parks and Older People's Sense of Community: Examining the Role of Urban Parks in Supporting Sustainable Development Goal 3 in Brisbane, Australia

**DOI:** 10.1111/ajag.70160

**Published:** 2026-04-17

**Authors:** Mahnoosh Hassankhani, Renee Zahnow

**Affiliations:** ^1^ Institute for Social Science Research The University of Queensland Brisbane Queensland Australia; ^2^ School of Social Science The University of Queensland Brisbane Queensland Australia

**Keywords:** healthy ageing, sense of community, social networking, urban parks

## Abstract

**Objective:**

This study examined the role of urban parks in fostering a sense of community among older people. This aligns with Sustainable Development Goal (SDG) 3, which emphasises supporting the environment to foster health and well‐being.

**Methods:**

We surveyed 123 individuals aged 70 years and older in Brisbane, Australia. Through the application of Structural Equation Modelling (SEM), we investigated the interplay between the attractions and barriers to parks and the sense of community and social networks.

**Results:**

Our findings showed that park visiting among older people is associated with a heightened sense of community. When parks have more attractive facilities, older adults visit more often. More attractive facilities are also associated with a heightened sense of community. However, we found no relationship between park facilities or park visits and social network size, although women reported larger local social networks than men.

**Conclusion:**

Our findings underscore the critical importance of parks for fostering a sense of community among older adults. These insights highlight the need for urban environments that are not only physically accessible but also perceived as safe, attractive and supportive of activity preferences.

## Policy Impact

This study directly informs urban planning policies by highlighting how quality urban parks enhance older adults' sense of community and social engagement, aligning specifically with Sustainable Development Goal 3 and target 3.4. Policymakers should prioritise accessible, senior‐friendly park facilities to promote healthy ageing, improve mental well‐being and reduce social isolation.

## 1 Introduction

Urban parks provide critical ecological functions, facilitate physical activity and contribute to a city's social infrastructure [1, 2, 3, 4]. The World Health Organization (2016) recommends that all people live within 300 m of green space, and emphasises that urban green spaces should be designed to be accessible to all population groups. Older people, as a result of retirement status, reduced daily obligatory mobility and, in some cases, due to physical ailments, tend to spend more time in their local communities compared to non‐local settings [5]. For this reason, urban parks offer accessible green space within the local neighbourhood and may be particularly pertinent for supporting the mental and social well‐being of older adults.

Sustainable Development Goal (SDG) 3, focussed on good health and well‐being, suggests that equitable access to healthy environments is essential to provide everyone with opportunities to live healthy, fulfilling lives at all ages. Studies show that for older adults, availability of social opportunities, such as public spaces within the residential neighbourhood, can influence health and well‐being [6] by providing opportunities for social support and fostering a sense of community [6, 7]. This is because the proximity to these social opportunities increases the likelihood of spontaneous encounters among community members, which are vital for a sense of community [1]. A sense of community can mitigate social isolation and loneliness [2, 3] and foster community support, trust and belonging [4, 5]. Thus, affluent areas with better access to public spaces also present greater opportunities to foster a sense of community than lower income areas, which results in unequal social outcomes.

Sense of community refers to the state of feeling interconnected with other community members [8, 9], has been described as feeling a sense of belonging and importance within the community [10] and is positively associated with physical and mental health [11, 12]. The literature suggests that sense of community is associated with heightened satisfaction with life and greater well‐being [3, 6], and leads to a sense of purpose and security [7]. In a comparative study between Australia and the United States (US), Sirgy et al. [8] suggested that, in both countries, sense of community is associated with satisfaction with life. Elgar et al. [9] studied this relationship in 50 countries using the World Values Survey (2005–2008) and concluded that a heightened sense of community positively correlated with life satisfaction, with stronger effects in countries with higher social capital. Guzman et al. [6] surveyed 4629 community members in Chile, and Zou et al. [10] surveyed 229 individuals in China; both found a direct and positive relationship between sense of community and satisfaction with life. These studies highlight the globally accepted importance of community in fostering public health and well‐being. For older people, sense of community is a mediator between age‐friendly initiatives and life satisfaction [11, 12, 13].

In Australia, the spatial logic of suburbs adversely affects community life and the sense of community [14, 15]. As parks go underfunded, shopping centres increasingly serve as accessible public spaces, social opportunities become ever more unevenly distributed, and older people find themselves negotiating a fragmented urban landscape shaped by market interests rather than their needs. While these processes affect many urban residents, their impact is particularly significant for older adults, who often rely more heavily on local social opportunities for connection, participation and visibility.

### 1.1 Urban Park for Healthy Ageing

Beyond being green spaces, parks present crucial social opportunities in local communities [16, 17, 18, 19]. Parks are among the few remaining urban spaces that connect residents to nature while fostering health, well‐being and social life [20]. The United Nations (UN) Sustainable Development Goal (SDG) 11 emphasises that green spaces (such as parks) can foster social inclusion and equity, promote health and well‐being, and support sustainable urban planning and design. The terminology used for parks is not consistent in the literature, and ‘park’ is sometimes used interchangeably with ‘urban green space’. To respect the terminology of the reviewed literature, we have retained the terms used in each source, which may result in some inconsistency in terminology in this section.

Given the changes in daily activities and reduced levels of mobility that can co‐occur with ageing, the local neighbourhood plays a key role in the everyday lives of older people [21]. As a result, local social opportunities, such as parks, are more significant in the everyday lives of older people in fostering their inclusion, visibility and autonomy [22]. As a result, local parks play a vital role in promoting healthy ageing [23] and ageing in place [24]. When parks are integrated into the everyday lives of older people, they transform from simple green spaces into meaningful places for socialising.

### 1.2 The Health and Social Outcomes of Visiting Parks

Visiting parks is important for sustaining social life and agency in older age [22]. However, measuring the factors that impact patterns of visitation is complex. Most commonly, studies focus on accessibility as a measure of visitation opportunity. Accessibility has been measured in different ways in the literature [15, 25, 26, 27] with physical proximity being the most common measure of park accessibility. Therefore, accessibility is operationalised as an objective spatial measure of physical proximity (e.g., networked or Euclidean distance), visit potential or area‐level density (e.g., the total amount of park available near the residence) or as a characteristic of the residential neighbourhood (e.g., mixed‐land use, percentage park). Less often, studies measure physical accessibility by assessing connectivity and public transportation options (Table 1) [50, 51, 52, 53].

**TABLE 1:** The key factors affecting park visitation, along with their conceptual definitions and relevant references.

Factor	Conceptualisation of accessibility	References
Physical proximity	The shortest distance is based on the road network between older adults' residences and the gate of the park	Enssle and Kabisch [28]; Jennings and Bamkole [29]; McCormack et al. [30]; Mohammadi Tahroodi and Ujang [31]
Road network distance	The distance along the road network from an individual's residence to parks	Groenewegen et al. [32]; Mohammadi Tahroodi and Ujang [31]; Wang et al. [33]
Availability of facilities	Various features and facilities are provided for the convenience, comfort, and enjoyment of park visitors	Ayala‐Azcárraga et al. [34]; Burnett et al. [35]; Enssle and Kabisch [28]; Groenewegen et al. [32]; McCormack et al. [30]; Wang et al. [27]
Size and availability of the park	The physical area or the amount of land the park covers	Ayala‐Azcárraga et al. [34]; Maas et al. [36]; McCormack et al. [30]
Maintenance quality	The upkeep and cleanliness of the park	Akpinar et al. [37]; Burnett et al. [35]; Hoffimann et al. [38]; McCormack et al. [30]; Zhai and Baran [39]
Traffic safety	The safety of an environment concerning vehicular traffic and perceived risk can deter people from accessing or using a space, affecting use	Bivina et al. [40];
Urban density	Indicates the concentration of buildings and population	Sun et al. [41]; Ye et al. [42]
Socioeconomic status	Refers to economic conditions that may affect access to amenities and resources, including the park	Dai [43]; Enssle and Kabisch [28]; Fan et al. [44]; Hoffimann et al. [38]
Transportation	Refers to the specific method of transportation that people use to travel, such as walking, biking, driving or using public transportation	Chang and Liao [45]; Fan et al. [44]; Jennings and Bamkole [29]
Landscape and design	Refers to the natural and built elements that shape parks' physical appearance and character, including topography, vegetation, water bodies, and other natural features, as well as the built environment	McCormack et al. [30]; Wang and Rodiek [46]; Yuan et al. [47]
Personal mobility and health	Encompasses the physical capability and means by which older adults move, whether independently or with assistance	de Keijzer et al. [22]
Perceived safety	When discussing park visits, crime safety can affect how comfortable and confident people feel about using a particular space	McCormack et al. [30]; Subramanian and Jana [48]; Vitman‐Schorr et al. [49]
Social interaction	Opportunities to engage and connect with others in the community, including family, friends, and new acquaintances	Mohammadi Tahroodi and Ujang [31]

Older adults may perceive accessibility differently from younger adults due to lifestyle differences, mobility limitations, personal safety concerns, experiences and expectations [54]. This aligns with SDG 3's objective to promote healthy lives and well‐being at all ages, as ensuring accessible parks for older adults can facilitate physical activity, reduce the risk of mental health issues and support active ageing.

The method we used for this purpose was a quantitative, cross‐sectional analysis focussed on the interaction of older people with their local parks in Brisbane. It explored the social value of parks by measuring the sense of community and social networks among older people in relation to accessibility to parks.

We collected primary data using a survey tool developed from previous studies. The survey tool contained three sections of questions. The first captured data on accessibility to parks and visits to examine the association between accessibility, use and social benefits. Although accessibility is often measured by physical distance alone [50], it is also a self‐report concept that can be affected by personal characteristics. Table 1 shows a summary of the literature on the non‐spatial factors that influence the perception of accessibility. To gain a comprehensive understanding of accessibility, the survey asked questions about safety, parks' amenities, landscape, maintenance and self‐reported proximity in minutes, adjusting the perception of closeness to the physical capacity of individuals.

Despite this growing literature, important gaps remain. Most studies of older adults and urban green space focus on physical and mental health outcomes, giving less attention to community‐level social outcomes such as sense of community and local social networks. Existing research also tends to examine either objective measures of park accessibility (e.g., distance‐based metrics) or perceived park quality and barriers, rather than considering how these dimensions work together to shape older people's use of parks. In addition, there is limited evidence from low‐density, car‐oriented cities such as Brisbane, where neighbourhood parks may be among the few everyday public spaces available to older adults. This study addressed these gaps by analysing how objective (neighbourhood park area) and perceived dimensions of park accessibility, including self‐reported proximity and park quality, are associated with park visits, sense of community and social networks among older people in Brisbane, Australia, interpreted through a *Right to the City* lens.

### 1.3 Conceptual Framework

In this paper, we approached the relationship between parks and sense of community for older people as an issue of urban justice through the lens of the *Right to the City*. This offers a useful tool for understanding older people's access to and use of urban parks as a question of urban justice rather than simply service provision [55, 56, 57]. Building on Lefebvre's (1968) conceptualisation, the *Right to the City* foregrounds residents' collective right not only to access urban spaces but also to appropriate and shape them through everyday practices, challenging urban forms and policies that prioritise market logics over lived experience [55, 58]. In this study, we understood the *Right to the City* for older adults through interconnected dimensions of distributive, participatory and recognitional justice, asking who has physical and perceived access to parks, who can be visibly and routinely present in these spaces and whose needs and preferences inform park design and governance [59]. From this perspective, parks are not neutral amenities but key social infrastructures through which older people claim visibility, belonging and a sense of community; unequal quality, accessibility or usability of parks therefore translates into unequal opportunities to enact the *Right to the City* in later life [60, 61]. By examining how park attractions and barriers shape visits, sense of community and social networks, our findings speak to broader debates on spatial justice and age‐friendly cities, highlighting the need for urban environments that support older adults' rights to participate in, benefit from and subtly reshape the public spaces that structure their everyday lives.

Our study strengthens SDG 3 by examining the extent to which access to quality urban parks can contribute to the health and well‐being of older people through community integration and support, as measured by their sense of community and social network. We examined two main hypotheses: (1) Park attractions, perceived barriers and proximity to parks (self‐reported walking time) will be associated with older adults' visits to parks (frequency and duration of visits); and (2) greater visits to parks will be positively associated with sense of community and social networks, with additional direct effects from park attractions and barriers on these community outcomes.

In this paper, we use urban parks as an umbrella term for publicly owned, publicly accessible parks within the Brisbane metropolitan area, and neighbourhood parks (or *local parks*) to refer specifically to small, walkable parks embedded in residential areas. Neighbourhood parks typically serve a local catchment within roughly a 10‐ to 15‐min walk from home and provide everyday opportunities for informal recreation and social interaction, in contrast to larger district or regional parks that draw visitors from across the city and often include specialised sports or destination facilities [21, 22, 51]. Our empirical focus was on these neighbourhood parks, because they are the most relevant everyday social infrastructure for older people who spend more time in their immediate communities.

## 2 Method

### 2.1 Data

**FIGURE 1:**
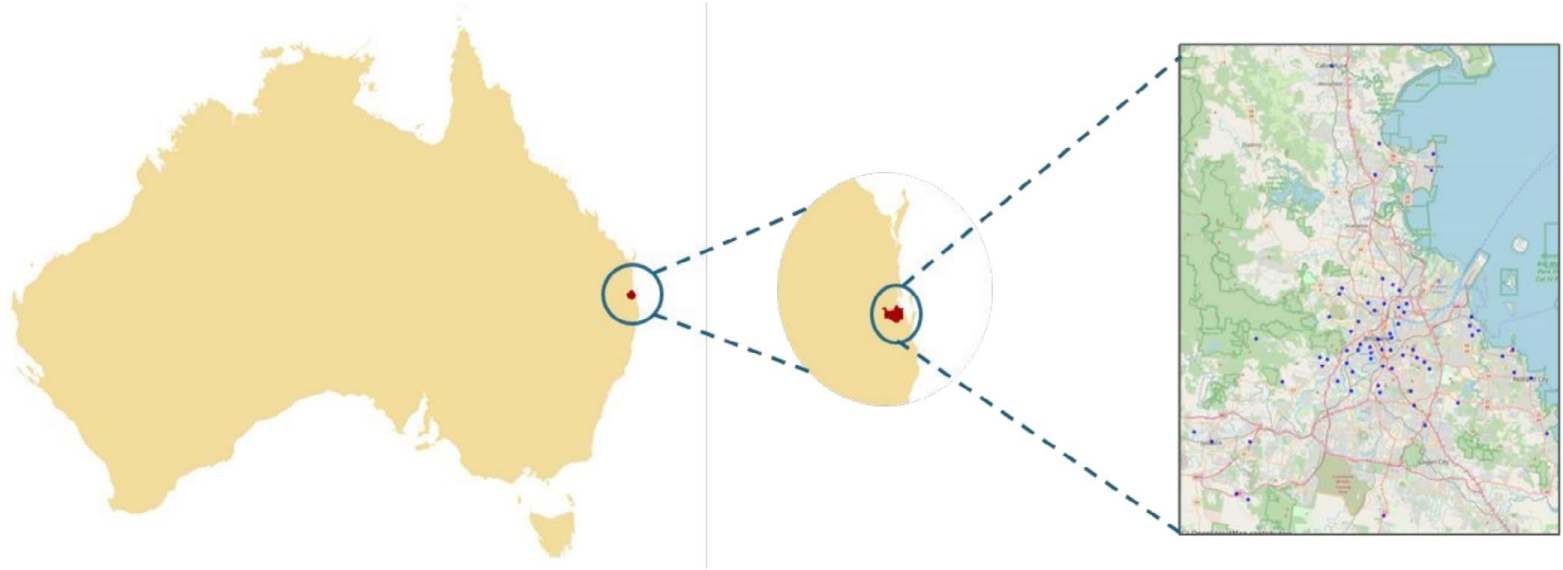
Shows the research area and the spatial distribution of participants across the Greater Brisbane area, Australia.

This study was conducted in the Greater Brisbane area, Australia (Figure 1). Brisbane is one of the greenest cities in Australia, with 2170 urban parks city‐wide. The primary data for this study came from the survey of 123 individuals over the age of 70 years residing in the Brisbane Greater Area in November and December 2023. Ethics approval for this study was obtained from the University of Queensland, Australia, Human Ethics Committee in November 2023 (approval no. 2023/HE001888). All participants were provided with a study information statement explaining the purpose of the research, what participation involved, that participation was voluntary, and that they could skip questions or withdraw at any time prior to submitting the survey without consequence. Informed consent was obtained prior to participation (for online and QR‐code responses, consent was recorded electronically before the survey commenced; for paper surveys, consent was indicated by return of the completed questionnaire; and for intercept recruitment, consent was obtained in writing before the survey). Responses were de‐identified and stored securely at the relevant institute's Data Manager system, and reported only in aggregate form.

We employed three recruitment methods: (1) convenience sampling via face‐to‐face intercept survey in public spaces and convenience sampling through flyers and third‐party stakeholders. For the online recruitment method, the survey link or QR code was shared on flyers distributed among older adults by community gatekeepers and posted on social media platforms frequented by older adults, such as Facebook pages. Individuals were provided the option to request a printed survey with a pre‐stamped and pre‐addressed envelope. In addition to our survey, we drew on two publicly available datasets to compute our variables: The Brisbane City Council Parks dataset available on their website (Brisbane City Council, 2020; data.brisbane.qld.gov.au) and the Australian Bureau of Statistics Census 2021.

### 2.2 Variables

#### 2.2.1 Dependent Variable

##### 2.2.1.1 Sense of Community

Sense of community was measured using five survey items. Participants were asked to rate their agreement on a 5‐point Likert scale (1 = strongly disagree to 5 = strongly agree) with the following statements: ‘People here and I care about similar things’; ‘Being in this community helps me meet my important needs’; ‘This community does a good job meeting members’ needs'; ‘Belonging to this community makes me feel positive’; and ‘I trust the people in this community’. These items were adapted from the sense of community scale developed by McMillan and Chavis [62] and capture core dimensions of the construct that are particularly relevant in later life, including shared values, fulfilment of needs and emotional attachment to the community. A brief five‐item version was used to reduce survey burden for older participants while retaining coverage of the main theoretical facets of sense of community. In this sample, the scale showed acceptable internal consistency and convergent validity (Cronbach's α ≈ 0.79; composite reliability ≈ 0.80; AVE ≈ 0.56), indicating that these items provide a reliable and valid indicator of sense of community.

##### 2.2.1.2 Social Networks

Social networks were assessed using five survey items. Participants rated their agreement or frequency on a 5‐point response scale for the following: ‘There is always someone I can talk to about my day‐to‐day problems’; ‘How often do you meet any of your neighbours?’; ‘There are plenty of people I can lean on when I have problems’; ‘I attend any public meeting in which there is a discussion of local affairs’; and ‘In the last 2 weeks, approximately how many people did you talk to about anything related to your personal life?’ Responses were coded so that higher scores indicated larger and more supportive local networks. These items were informed by the social network measures proposed by Lubben et al. [35] and were selected to capture both structural aspects of networks (frequency and number of contacts) and functional aspects (perceived availability of emotional and practical support) that were important for older adults. The resulting five‐item scale demonstrated acceptable reliability and convergent validity in our confirmatory factor analysis (Cronbach's α ≈ 0.70; composite reliability ≈ 0.79; AVE ≈ 0.52), supporting its use as a concise but theoretically grounded measure of older people's social networks.

#### 2.2.2 Independent Variables

##### 2.2.2.1 Proximity to Parks

Proximity to parks was measured using a self‐reported walking‐time question. Participants were asked how long it usually took them to walk from home to the park they visited most often (1 = < 5 min; 2 = 5–10 min; 3 = 10–20 min; 4 = 20–30 min; 5 = more than 30 min). Lower scores, therefore, indicated proximity to a preferred park. In addition, we calculated the proportion of land in each participant's residential neighbourhood that was classified as a public park using Brisbane City Council land use data. This park area variable was used descriptively to characterise neighbourhood green space but was not included in the structural equation model (Table A1).

##### 2.2.2.2 Park Characteristics (Attractions and Barriers)

The park characteristics score was created by aggregating survey responses on a 5‐point Likert scale for items such as safety, maintenance, pet‐friendliness and the variety of amenities they have. These responses were averaged to provide an overall measure of park characteristics, ensuring equal weighting of each item. Aggregation allowed for a comprehensive representation of participants' experience of the park characteristics, focussing on the local parks they regularly visit. This informed the measure of accessibility by providing insight into the level of comfort parks provide and how well they address the needs of older people and encourage visits. Table 2 shows the variables we used in detail.

**TABLE 2:** Descriptive table.

	Mean	SE	95% confidence interval		Median	SD
			Lower	Upper		
Variety of amenities	3.56	0.10	3.37	3.75	4.00	1.04
Pet‐friendly features	3.81	0.06	3.69	3.94	4.00	0.69
Maintenance and cleanliness	3.92	0.07	3.77	4.06	4.00	0.81
Landscape features	3.86	0.07	3.73	4.00	4.00	0.77
Traffic‐safety	2.55	0.09	2.37	2.73	2.00	1.00
Crossing a dangerous road	2.81	0.10	2.62	3.01	3.00	1.09
Safety of pathways	4.00	0.08	3.85	4.15	4.00	0.85
Frequency of visit	5.75	0.22	5.32	6.19	6.00	2.41
Length of visit	2.89	0.13	2.62	3.15	3.00	1.41
Recognising people in the community	3.68	0.09	3.50	3.86	4.00	1.01
Experience of exclusion	4.34	0.08	4.18	4.5	5.00	0.9
Trust community	2.67	0.11	2.46	2.88	3.00	1.16
Feeling close to the community	3.44	0.09	3.26	3.62	4.00	1.01
Participating in community events	2.26	0.10	2.07	2.45	2.00	1.06
Sense of belonging to the community	3.66	0.09	3.49	3.83	4.00	0.94
Number of social contacts in the community in the last 2 weeks	1.43	0.11	1.21	1.65	1.00	1.22
Experience of discrimination	1.59	0.08	1.44	1.75	1.00	0.88
Sense of isolation	2.02	0.09	1.85	2.19	2.00	0.95
Level of disability	2.36	0.07	2.22	2.49	3.00	0.73
Age	2.10	0.10	1.91	2.29	2.00	1.05
Safety	3.99	0.09	3.82	4.16	4.00	0.93
Area of parks in the neighbourhood	3.43	0.07	3.30	3.57	4.00	0.73
Number = 123						

Park attractions and park barriers were modelled as separate constructs because they capture conceptually different aspects of the park experience for older adults. Attractions reflected positive qualities that draw people into parks (e.g., amenities, maintenance, safety and features that meet diverse needs), whereas barriers captured perceived obstacles that may discourage or prevent visits (e.g., unsafe pedestrian conditions, the need to cross major roads, feelings of insecurity). An older person can experience high attractions and high barriers at the same time (e.g., a well‐equipped park that nonetheless requires crossing a busy road), so we did not treat these as opposite ends of a single continuum. Because attractions and barriers are conceptually related, we examined potential collinearity before estimating the SEM.

The attraction and barrier items were selected a priori to represent the park quality and accessibility domains that literature repeatedly links to park visitation and the activities that parks can foster, especially for older adults [27, 30, 49]. These items mapped onto the identified determinants of park use:

- amenities and supportive facilities (e.g., seating, toilets, shade and activity features) [27, 30, 34];
- maintenance and cleanliness [37, 38, 39];
- aesthetics and landscape; and [34, 46]
- perceived safety (including concerns about antisocial behaviour and personal security) [30, 48, 63].

They also mapped to pedestrian and traffic‐related barriers [27, 39, 40] that can constrain older adults' willingness to reach and use parks (e.g., unsafe crossings, traffic exposure and poor paths) [27, 40, 48]. These factors aligned with the variable of park attributes associated with the tendency to use and visit parks [30, 64] and with age‐friendly frameworks that highlight clean, well‐maintained parks, adequate outdoor seating and safe pedestrian infrastructure as priorities for supporting older people's outdoor mobility and engagement [13, 61, 65].

##### 2.2.2.3 Demographic Information

Control variables were included to account for demographic factors known to influence park visits, sense of community and life satisfaction among older adults: sex (0 = male, 1 = female); age category (1 = 70–74 years, 2 = 75–79 years, 3 = 80–84 years, 4 = 85–89 years, 5 = 90–94 years, 6 = 95+ years); and disability status (1 = No, 2 = Yes, to some extent, 3 = Yes, a lot).

#### 2.2.3 Analysis

To examine how park characteristics and proximity to parks relate to older people's visits to parks, sense of community and social networks, we created a structural equation model (SEM) for its capability to examine complex relationships and latent variables. While other regression models can also provide insight into these relationships, SEM can better estimate direct and indirect pathways and measure the influence of mediating factors. For example, while park accessibility may be associated with a stronger sense of community among older adults, it may be a sense of community rather than park accessibility that is the more proximate indicator of life satisfaction. A SEM is also an appropriate option for this research because it can handle latent constructs and manage complex data structures. The robustness of the models can be observed through the Comparative Fit Index, Tucker–Lewis Index, Root Mean Square Error of Approximation, Standardised Root Mean Square Residual and confirmatory factor analysis. Table 3 summarises studies that employed SEM for a similar purpose to the present study—to explore complex relationships between individuals and the environment—highlighting some key points from each. The structural model can be formally expressed as:

η=Bη+Γξ+ζ

Here, η represents latent endogenous variables, B captures their interrelations, Γ reflects the impact of observed exogenous variables (ξ), and ζ is the residual error. All models were conducted in RStudio using the packages ‘lavaan’, ‘sem’ and ‘semplot’.

**TABLE 3:** Summary of a review of literature that used SEM for a similar purpose.

References	Aim
Allard‐Poesi et al. [66]	Model the influence of nature perceptions near urban residences on well‐being
Chen et al. [67]	Examine the correlation of environment, usage patterns, and restoration in rooftop gardens
Guo et al. [68]	Explore the pathway from the built environment to mental and subjective well‐being
Hong et al. [69]	Study how frequency and time spent in parks affect well‐being
Lai and Deal [70]	Analyse happiness and well‐being in urban parks
Yang et al. [71]	Examine mediators of green space‐mental health associations among migrants
Zhang et al. [72]	Examine links between self‐reported health, attachment to park, and green space availability
Zhou et al. [73]	Investigate the dynamic relationship between blue‐green spaces and mental restoration

In the structural part of the SEM, park attractions, park barriers, proximity to parks and the demographic controls (age, sex and disability) were specified as exogenous variables. Visits to parks, constructed from the frequency and duration of visits, were specified as an endogenous mediator predicted by park attractions, park barriers, proximity to parks and the demographic controls. Sense of community and social networks were modelled as latent endogenous variables with five indicators each, and were predicted by park attractions, park barriers, proximity to parks, visits to parks and the same demographic controls. This specification allowed us to estimate both the direct associations of park characteristics and proximity with sense of community and social networks, and the indirect effects operating through visits to parks.

Most of the survey indicators (e.g., sense of community, social networks, park attractions and barriers) were measured on 5‐point Likert scales and showed departures from multivariate normality. To account for the ordinal nature of these items, we estimated the measurement and structural models using a robust diagonally weighted least squares estimator with mean‐ and variance‐adjusted test statistics (WLSMV) in the lavaan package in the statistical package R. This estimator is recommended for SEM with ordinal indicators because it models the polychoric correlations between items and produces robust standard errors and fit indices under non‐normality. For comparison, we also estimated the model using maximum likelihood (ML); the pattern, size and significance of the key paths were substantively unchanged, so we report the WLSMV results here.

## 3 Results

**FIGURE 2:**
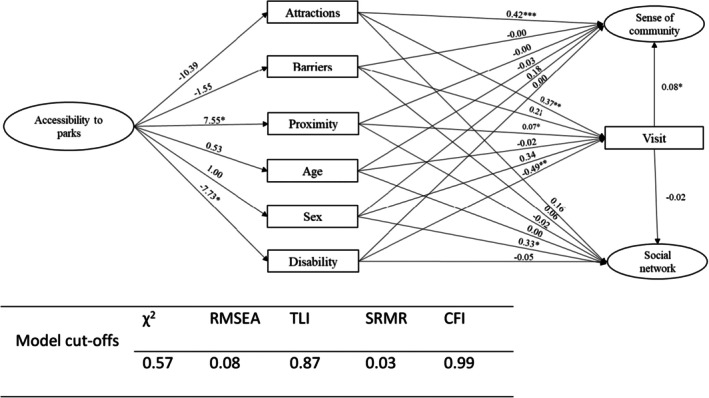
Structural equation model of associations between park characteristics, visits to parks, sense of community and social networks. Arrows represent standardised regression paths (β) between variables, estimated simultaneously while controlling for age, sex and disability. Sense of community and social networks are modelled as latent variables; factor loadings are omitted for clarity. All coefficients are standardised regression coefficients (β) from the structural part of the SEM. Sense of community and social network are estimated as latent variables with multiple indicators; their factor loadings are not shown here but were all positive and statistically significant in the measurement model.

The SEM results indicated that park attractions and proximity to parks were positively associated with visits to parks, whereas disability was negatively associated with visits. Higher attraction scores were related to more frequent and longer visits (β = 0.37, SE = 0.14, *p* < 0.01), and shorter self‐reported walking time to the nearest park was also associated with more visits (β = 0.07, SE = 0.03, *p* < 0.05). In contrast, higher levels of disability were associated with fewer visits (β = −0.49, SE = 0.17, *p* < 0.01). In turn, both park attractions (β = 0.42, SE = 0.10, *p* < 0.001) and visits to parks (β = 0.08, SE = 0.04, *p* < 0.05) were positively associated with sense of community, while proximity to parks, barriers, age, sex and disability showed no significant direct effects on sense of community. None of the park‐related variables showed statistically significant associations with social networks, although being a woman was associated with larger social networks (β = 0.33, SE = 0.15, *p* < 0.05) (Table 4; Figure 2).

**TABLE 4:** The results of the SEM model (Source: Data collected and analysed by authors, 2023).

	Estimate	SE	*z* value	CI 95%
Visit				
Park attractions	0.37**	0.14	2.58	[0.09, 0.65]
Park barriers	0.21	0.13	1.62	[−0.04, 0.46]
Proximity	0.07*	0.03	2.08	[0.004, 0.136]
Age	−0.02	0.11	−0.18	[−0.24, 0.20]
Sex	0.34	0.24	1.42	[−0.13, 0.81]
Disability	−0.49**	0.17	−2.88	[−0.83, −0.15]
Sense of community				
Park attractions	0.42***	0.1	4.20	[0.22, 0.62]
Park barriers	−0.00	0.05	0.00	[−0.10, 0.10]
Visit	0.08*	0.04	2.00	[0.00, 0.16]
Proximity	−0.00	0.04	0.00	[−0.08, 0.08]
Age	−0.03	0.04	−0.75	[−0.11, 0.05]
Sex	0.18	0.10	1.80	[−0.02, 0.38]
Disability	0.00	0.08	0.00	[−0.16, 0.16]
Social network				
Attraction	0.16	0.15	1.07	[−0.13, 0.45]
Barriers	0.06	0.08	0.75	[−0.09, 0.21]
Visit	−0.02	0.05	−0.40	[−0.12, 0.08]
Proximity	0.00	0.06	0.00	[−0.12, 0.12]
Age	−0.04	0.06	−0.67	[−0.16, 0.08]
Sex	0.33*	0.145	2.28	[0.05, 0.61]
Disability	−0.05	0.11	−0.45	[−0.27, 0.17]

*Note:* *p < 0.05, **p < 0.01, ***p < 0.001.

## 4 Discussion

This study highlighted four key findings. First, attractions and proximity were associated with older adults' park visits, while disability was associated with fewer visits. Second, park attractions and park visits were positively associated with sense of community, with park attractions showing the stronger association. Third, park barriers and proximity were not directly associated with sense of community once park visits and park attractions were considered. Fourth, park attractions, barriers and proximity were not significantly associated with social networks among older people in our sample size, although women reported larger social networks than men.

First, in partial support of our first hypothesis, park attractions and proximity to parks were both important indicators of how often and how long older adults visit parks. This finding is consistent with studies showing that older adults' use of parks is shaped by features such as amenities, maintenance and safety, as well as proximity to the park [30, 34, 39, 74, 75]. Our results suggest that park features do not operate uniformly, such that attraction and proximity were associated with visitation whereas barriers were not significant in the model. This does not mean that barriers are not important; instead, it may indicate that positive qualities and convenience play a more significant role in shaping frequency and duration of visits to the park, while barriers may operate more strongly in park choice, route negotiation, or among less mobile groups. Older adults may continue to visit parks when supportive features are present, even where some constraints remain. This reinforces the importance of maintaining everyday park usability (e.g., seating, shade, paths, upkeep, and perceived safety) for sustaining regular visitation in later life [30, 34, 39, 75].

Second, the association between park attractions, park visits and sense of community highlight the significance of the surrounding environment to foster social life of individuals in later life [11, 12, 13, 15, 29]. The contribution here is not simply that parks are beneficial but that the social value of neighbourhood parks appears to depend on whether they are experienced as attractive and supportive of everyday activities in later life. At the same time, the absence of a direct association between proximity and sense of community in the SEM suggests that living closer to a park is not sufficient on its own; repeated use and positive park experience appear to matter more for community attachment. These findings inform urban policies and age‐friendly city agendas (such a WHO age‐friendly city agenda, [76]) and explains provision is not sufficient if parks do not offer the features that provide comfort and attraction for visits. Without these features, parks cannot turn into social destinations and remain as green areas that are parts of the built environment in the everyday lives of older people.

Third, the disability finding is especially important for interpretation through an urban justice lens. Older adults with higher levels of disability reported fewer park visits, and because park visits were associated with sense of community, disability may indirectly reduce access to the social opportunities provided by local parks. This aligns with research showing that mobility and environmental constraints shape older adults' ability to use social opportunities [77, 78]. This finding indicates that older people are not a homogeneous group, and their specific characteristics (such as disability) can directly and indirectly shape their social practices and their relationship with and within their communities. Consideration of these differences is significant in creating communities that successfully support the social life and visibility of those older people with different needs and characteristics.

Fourth, park features, including attractions, proximity and barriers, were not associated with social network in later life, even though park attractions and visits were associated with sense of community. This finding is theoretically important and should not be interpreted as a null result as it suggests that neighbourhood parks may strengthen subjective community connection (e.g., familiarity, trust, belonging and local attachment) without necessarily increasing the number of local social connections measured by the social network. This distinction is consistent with research showing that parks can support casual, low‐threshold social contacts without necessarily expanding personal networks [18].

This study contributes to the literature and makes a distinction between provision, use and social benefits. Although the literature increasingly underpins the social benefits of parks in later life [16, 19, 79], our findings suggest that provision must be associated with the features that support social engagement in later life and offer the features that create attraction and promote longer and more frequent visits. By interpreting these findings through the lens of the *Right to the City* and in relation to SDG 3, we highlight the justice implications of unequal access to attractive, usable local parks in later life and identify neighbourhood parks as a key site for age‐friendly urban policy in Australia.

### 4.1 Limitations

The accuracy of SEM requires large datasets, whereas the sample in this survey is relatively small for this method. To address this, we kept the model theoretically grounded and structurally simple and considered multiple model fits to select the best structure. We are also aware that this approach cannot fully mitigate the impact of a small sample size; it is worth mentioning that the findings of SEM in this study have been interpreted as exploratory, rather than definitive causal effects. The aim was to test plausibility and internal coherence, consistent with critical realism, which permits inferences about causal mechanisms, even in small contexts where theory and empirical structures align.

Another important limitation is related to data collection. Distributing the surveys through gatekeepers and in public places risks excluding those who are not socially active or physically mobile. However, online strategies can mitigate the effect of this sample bias.

## 5 Conclusions

Social isolation and loneliness are among the most significant conditions that threaten the health and well‐being of older people across the world, and Australia is no exception; however, the opportunities for older people's visibility and active participation in the community can empower them to tackle these conditions. Inclusive urban living stands as a central focus across age‐friendly research and policy, emphasising the importance of justice to promote healthy ageing [65]. This study extends the urban justice discourse and explains that urban policies need to go beyond the equal provision of services and consider the features that make parks usable and more than mere green spaces, which are part of the built environment. We suggest going beyond one‐size‐fits‐all design, considering participatory planning as an effective approach to identifying the needs of older people more precisely and creating local social opportunities with their partnership. We recommend that future research employ longitudinal, multi‐wave surveys with older adults in community settings to better understand the complex relationships between visits to parks and a sense of community.

## Funding

The authors have nothing to report.

## Ethics Statement

This study received ethics approval from the University of Queensland, Australia, Human Ethics Committee in November 2023 (approval no. 2023/HE001888).

## Conflicts of Interest

The authors declare no conflicts of interest.

## Data Availability

Research data are not shared.

## Appendix A

**TABLE A1:** Variables to measure attractions and barriers to visit (independent variables).

Question	Answers	Survey
How much park can you access within 10–15 min from your home?	○ None ○ A little/less than other places ○ Some/similar to other places ○ A lot/more than other places ○ Not sure	Adapted from the PHENOTYPE survey and informed by public engagement group
I feel safe to access local parks and spend time there	○ Strongly disagree ○ Disagree ○ Neither agree nor disagree ○ Agree ○ Strongly agree	Adapted from the PHENOTYPE survey Used in the most recent waves of Monitor of Engagement with the Natural Environment survey
There are parks that are accessible to me and provide a variety of amenities	○ Strongly disagree ○ Disagree ○ Neither agree nor disagree ○ Agree ○ Strongly agree	Adapted from Monitor of Engagement with the Natural Environment survey
The parks in my neighbourhood are pet friendly	○ Strongly disagree ○ Disagree ○ Neither agree nor disagree ○ Agree ○ Strongly agree	Adapted from the Blue Health Survey
I am satisfied with the maintenance and cleanliness of the parks in my community	○ Strongly disagree ○ Disagree ○ Neither agree nor disagree ○ Agree ○ Strongly agree	Adapted from the PHENOTYPE survey
Pedestrian pathways to my local parks require crossing busy roads or major highways	○ Strongly disagree ○ Disagree ○ Neither agree nor disagree ○ Agree ○ Strongly agree	Adapted from Perceived behavioural control survey
Pedestrian pathways to my local parks can be unsafe to access	○ Strongly disagree ○ Disagree ○ Neither agree nor disagree ○ Agree ○ Strongly agree	Adapted from Perceived behavioural control survey
Generally, how easy is it for you to get to parks in your community?	○ Very easy ○ Easy ○ Neither easy nor difficult ○ Difficult ○ Very difficult	Adapted from Perceived behavioural control survey
How far do you think is the closest public park to your home?	_____ Minute walk	Adapted from the Monitor of Engagement with the Natural Environment survey
How often do you usually visit or pass through parks for any reason?	○ Never ○ Once a year ○ Once every 3 months ○ Once a month ○ 2–3 times a month ○ Once a week ○ 2–3 days a week ○ 3–5 days a week ○ 6–7 days a week	Based on Monitor of Engagement with the Natural Environment Survey and Welsh Outdoor Recreational Survey
On each visit, approximately how long do you spend in the parks?	○ < 5 min ○ Between 5 and 30 min ○ 30–60 min ○ 1–2 h ○ 2+ h	Adapted from Monitor of Engagement with the Natural Environment survey
